# A Warning to the Profession

**Published:** 1884-01

**Authors:** A. W. Freeman

**Affiliations:** 125 State Street, Chicago, Ill.


### A WARNING TO THE PROFESSION.

125 State Street, Chicago, Ill.,

December 15th, 1883.

*Editor Independent Practitioner:*

Near evening of March 11th, 1882, while standing at the Morrison dental chair, pain and swelling of the right leg from knee to foot first began.

In a few hours the pain was excruciating and the least touch of the knee unbearable.

My usual physician was in Europe, and a medical Professor of good reputation came in his stead. He thought the trouble was inflammatory rheumatism, and for that instituted treatment. After a couple of weeks I was able to be up, and the soreness and swelling was so far modified that I began professional work, sitting upon a dental stool. As the enlargement of the knee diminished, a small swelling was apparent on the upper part of the tibia, near the joint.

My own doctor returning, he assumed the case, calling the ailment rheumatism. There was little change in the soreness and lameness of the knee up to August. I was then advised to spend a long vacation at Avon Springs, New York, taking hot sulphur baths, which it was hoped would be beneficial. I returned about the middle of September, no better, but with a slight increase of the tibial swelling.

Early in October I applied to a Professor of Surgery for his opinion of the case. "This swelling endangers the joint," said he. "The enlargement of the joint is probably secondary, and there is an effusion of synovial fluid about it. We will keep up the rheumatic treatment, and watch the swelling."

November 29th, it seemed to contain fluid and was aspirated, then carbolized, and shut up to heal by first intention. A week later it was again full, and was treated in the same way. At the end of the third week it was opened and a drainage tube inserted. The contents of the tumor each time were thick blood and a few flocks of matter. It was treated with carbolic solution by syringing twice a day. A ten pound weight was now attached to the foot to separate the joint, and traction continued three weeks.

February 26th, showing little change after nearly three months, I was put under gas, and kept under it, while examination was made to find the necrosed bone.

Again, April 2nd, ether was administered and a thorough exploration made along the front of the tibia for several inches, and down underneath to promixity of the large artery and nerve. These attempts were fruitless ; everywhere the bone was well covered with periosteum. The joint from the first inception of disease was partially stiff, and entirely so at times. The soreness rapidly increased after these operations, until the least jar or movement of the knee produced intensely sharp pain.

A second surgeon was now called for advice. He thought an operation desirable to make a stiff joint, and keep the limb. A third surgeon said it was too late for that operation, and advised that the limb be soon amputated. My own surgeon reluctantly concurred with this opinion, and took it off, June 14th, 1883.

The suffering of the first week was bad enough, but the agony of neuralgic pain for over three weeks following was terrific. The best soporifics were literally worn out, securing scarcely two hours rest out of the twenty-four. Tight bandaging and medicines were useless. Night and day I held the stump with both hands to prevent the fearful throes.

The lower end of the femur was found to be slightly softened. The cartilage was partly destroyed. The swelling about the knee was filled with pus, and synovial fluid. Between the tibia and fibula, underneath the knee, was a large sac of pus, which probably filtered down from the joint, and slowly escaped by the tibial swelling.

After calling the aid of a surgeon, I first recalled the fact that about noon of the same day the painful troubles began, I hit the knee against the end of the crank of the dental chair, producing a keen pain which lasted some seconds. I had often hurt it in the same way, but less severely. A year or more previously, I had received a severe blow on the limb, at or near the joint, from a partial revolution of the crank of the chair, the dog not catching firmly in the cog-wheel. I had taxed the knee for years with the entire running of Green's, then White's, dental engines, often with a feeling of discomfort which it was supposed was rheumatic.

Dr. J. F. Marriner of Ottawa, Ill., says the running of the engine has seriously affected his knee. Dr. W. O. Kulp, of Davenport, Iowa, was laid up two weeks from inflammation of the synovial membrane, caused by use of the engine. It was reduced by counter irritation.

A water motor, electricity, or an assistant, should run the engine. For the dentist to do it, is to expend nerve force or vitality which should be kept as future reserve power. The body cannot be overworked in brain or limb with impunity. It must have vacations for rest and recuperation, or like machinery for oiling and repairs. We should heed all warnings of its weakness.

Small injuries of the knee or its surrounding parts, may result in the loss of the limb. We cannot blame others for our own stupidity or carelessness. We well know that the true diagnosis of disease is sometimes very difficult, and with humility admit that often in mental darkness grope all they who attempt to repair any part of God's image.

In retrospect, I now consider that the many thumps, and the running of lathe and engine for over twenty years were preparatory aids, while the immediate cause of the primal inflammation of the knee was the severe blow upon the crank, given a few hours before twinging pain began.

Very truly yours,

A. W. FREEMAN.

